# Effect of Baduanjin exercise on sarcopenia in older adult patients: a multicenter, randomized controlled trial

**DOI:** 10.3389/fpubh.2025.1757862

**Published:** 2026-01-12

**Authors:** Li Luruihui, Xiao Jing, Tian Yuan, Liang Wenming, Tan Tianyang, Ji Yuxuan, Cao Bingyan, Zhang Hanzhi, An Danqiang, Zhang Yufan, Gao Peng, Wang Yuzhe, Lin Zhuying

**Affiliations:** 1 School of Clinical Medicine, Beijing University of Chinese Medicine, Beijing, China; 2 Xiyuan Hospital of China Academy of Chinese Medical Sciences, Beijing, China; 3 Physical Education Institute, Jimei University, Xiamen, China; 4 Life Science Center, Vilnius University, Vilnius, Lithuania

**Keywords:** Baduanjin, older adults, randomized controlled trial, sarcopenia, study protocol

## Abstract

**Background:**

Sarcopenia is an age-related syndrome characterized by muscle loss and reduced muscle strength, with a high incidence rate, significantly increasing the risk of falls, fractures, and death. Baduanjin, a traditional Chinese exercise, compared with traditional rehabilitation programs, has low intensity, high safety and strong interest, and is particularly suitable for patients who cannot tolerate high-intensity exercise. This study will evaluate the therapeutic effect of Baduanjin on sarcopenia.

**Method/design:**

This trial is a multicenter randomized controlled trial that will recruit 200 participants,and is randomized (1: 1) the subjects to the intervention group (Baduanjin + health education + dietary guidance, BHD) and the control group (health education + dietary guidance, HD). After enrollment, health lectures were given to all subjects. The intervention group received 12 weeks of Baduanjin intervention, with each class lasting 70 min, once a week, and provide online guidance for home workouts four times a week, while the control group maintained their original exercise habits. Health education and dietary guidance were also provided. Observation indicators will be tested before the trial begins and during the follow-up period after the intervention ends.

**Discussion:**

This trial is a randomized controlled trial that will evaluate the intervention effect of Baduanjin on sarcopenia. According to the study of this trial, it will provide evidence for Baduanjin to improve sarcopenia.

**Clinical trial registration:**

ITMCTR2024000887.

## Background

Sarcopenia is an age-related geriatric syndrome characterized by reduced muscle mass, decreased muscle strength, reduced physical function, and decreased ability of daily living (1). The disease affects about 14 to 33% of people aged 65 and above (2). It seriously endangers the health of older adults, not only leading to a decline in their quality of life, but also increasing the risk of adverse consequences such as falls, fractures and death, causing a heavy social and economic burden (3–6).

Sarcopenia has a complex pathogenesis (7, 8). Generally speaking, it is a type of age-related disease caused by multiple factors working together. It is manifested in the reduced physical activity capacity and decreased exercise volume of older adults (1). At present, the internationally recognized treatment method is aerobic exercise, resistance exercise combined with nutritional support (9–12). However, it faces multiple restrictive factors during the implementation process,such as requirements for patients’ basic physical fitness, poor compliance with training forms, and insufficient standardization of exercise prescription implementation in primary medical institutions. Baduanjin is a traditional Chinese guiding exercise with high recognition among Chinese people. It is moderate in intensity, entertaining, safe and effective, easy to learn, and is a feasible treatment for sarcopenia (13–15). Current researches show that Baduanjin has a good effect on motor function (16–18). By combining low-intensity aerobic exercise with the transition of virtual-real gait, it simultaneously activates the core and lower limb muscle groups and strengthens proprioceptive and coordination. It can improve joint range of motion, stability and dynamic balance ability, reduce the risk of falls, and has a good effect on endurance, strength, balance and flexibility (14). In our previous clinical observation, it was found that Baduanjin enhances muscle strength in patients and improves their quality of life. In addition, Baduanjin exercises have less cardiopulmonary load and higher safety (19, 20), and are also suitable for patients with limited cardiopulmonary function or contraindications to exercise, which can effectively expand the range of people suitable for exercise rehabilitation.

However, there is currently a lack of large-sample clinical studies to verify the rehabilitation effect of Baduanjin on sarcopenia. To this end, a 20-week clinical randomized controlled trial (including a total of 12 weeks of intervention and a follow-up visit at the 20th week.) was designed to systematically evaluate the intervention effect of Baduanjin on older adult patients with sarcopenia through multiple indicators such as muscle strength, muscle mass and physical function.

## Methods

This study is designed and will be conducted and reported according to the Consolidation Standards of Reporting (CONSORT) 2010 Statement regarding randomized controlled trials (21).

### Study design

**Figure 1:**
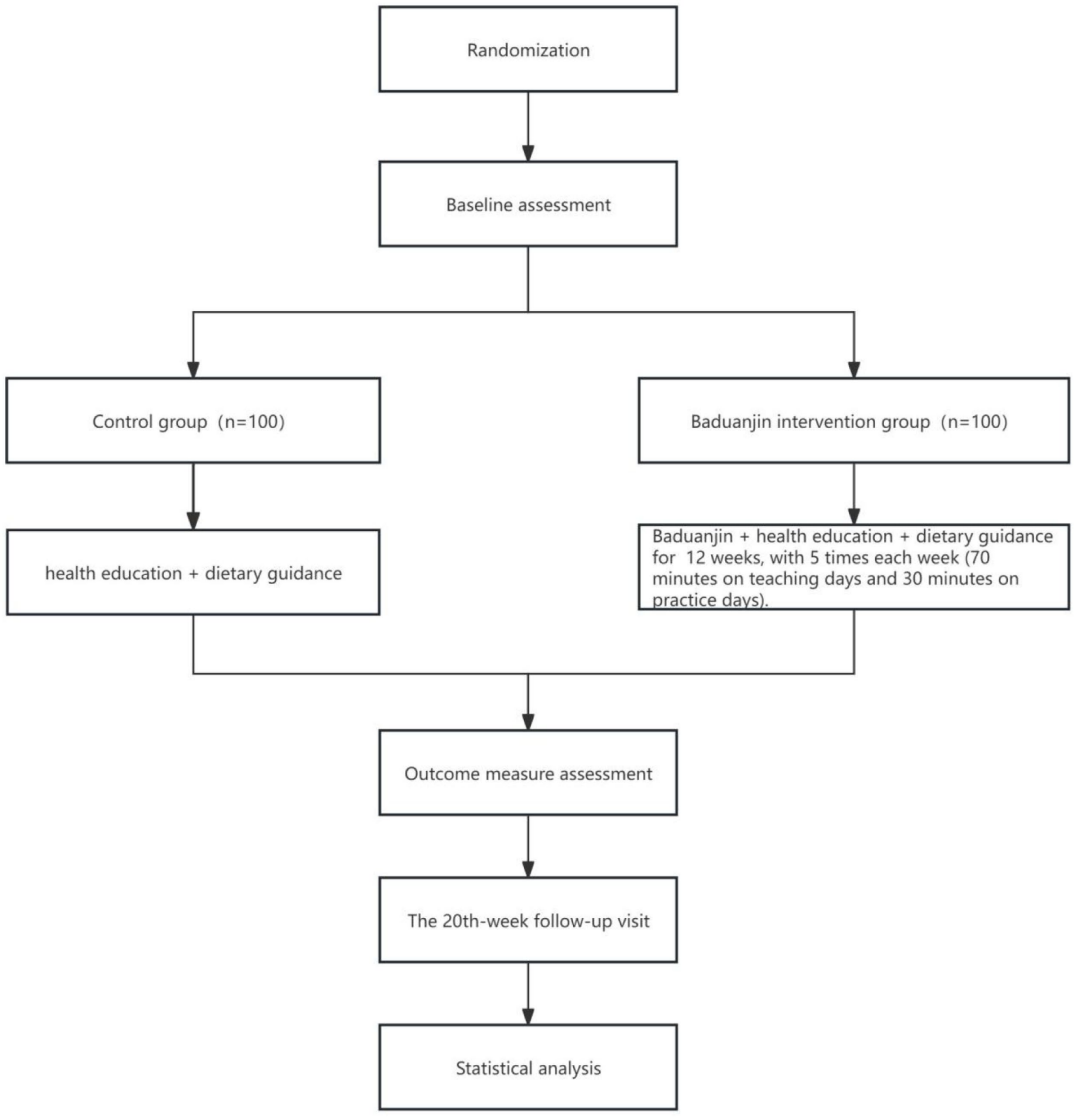
Trial flowchart.

**Figure 2:**
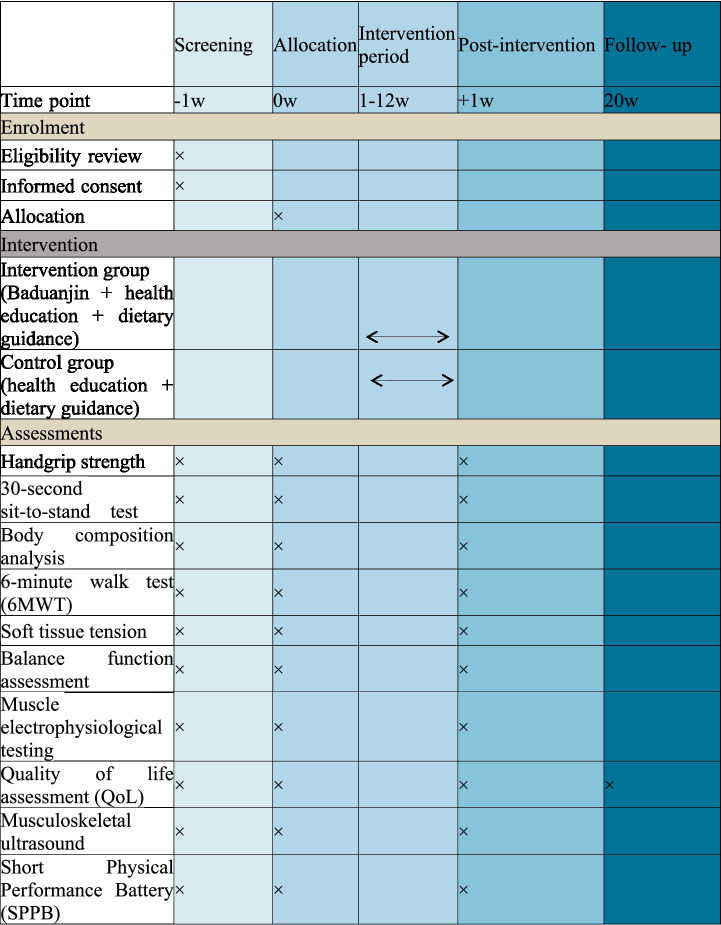
Schedule of enrollment, intervention measures, and assessments.

This study adopted a multicenter, randomized, controlled trial design to evaluate the rehabilitation effect of Baduanjin on sarcopenia compared with non-specific exercise (maintaining the original exercise routine without additional exercise intervention), involving a total of 200 patients diagnosed with sarcopenia. They were randomized (1:1) to the intervention group (Baduanjin + health education + dietary guidance, BHD) and the control group (health education + dietary guidance, HD), The trial procedure and outcome assessment schedule are presented in Figures 1, 2. The trial was registered on International Traditional Medicine Clinical Trial Registry on December 28, 2024 (Registration number ITMCTR2024000887).

### Sample size calculation

Revise it to read as follows:Sample size was calculated based on the inter-group independent sample mean comparison formula, the calculation is based on the research of Chang KV et al. (22) (The primary outcome measure was handgrip strength).Assuming a two-sided type I error (*α*) of 0.05 and a test power of 85% (type II error *β* = 0.15),it was calculated that 80 subjects were needed in each group (a total sample size of 160). Further considering a 20% dropout rate, ultimately 100 cases (200 cases in total) need to be recruited per group. Calculations were performed using G*Power software, version 3.1.9.7.

### Study population

#### Eligibility criteria

Diagnostic criteria (2): “Asian Working Group for sarcopenia: 2019 Consensus Update on Sarcopenia Diagnosis and Treatment” criteria for Possible sarcopenia, calf circumference (for men < 34 cm, for women < 33 cm), SARC-F scale ≥4 points, Handgrip strength (for men < 28 kg, for women <18 kg).

Inclusion criteria:

1. Conforming to the above diagnostic criteria;
2. 65 ≤ age ≤80;
3. Gender is not limited;
4. Be able to independently complete daily activities and basic communication, have no mental or neurological disorders, and be able to correctly understand the scale;
5. The individual or co-residents are proficient in using smart devices such as smartphones;
6. Voluntarily participate in the study and sign the informed consent.

Exclusion criteria:

1. Contraindications to exercise rehabilitation;
2. Those with severe liver or kidney diseases;
3. Patients with malignant tumors who are undergoing chemotherapy, radiotherapy or have bone metastases;
4. Those who are unable to complete the exercise treatment program due to sprains, fractures, severe joint deformities, etc.
5. Severe fracture osteoporosis with T value ≤ − 2.5SD and one or more brittle fractures at the same time.

### Recruitment

The subjects of this trial will be recruited through community promotion, hospital outpatient services, etc. The subjects are from Haidian District, Beijing, China; Taiyuan City, Shanxi Province, China; Tianjin City, China; Jining City, Shandong Province, China; Suzhou City, Jiangsu Province, China. After the researchers confirm the subjects’ willingness to participate and confirm their eligibility for inclusion, the informed consent process will be carried out. After signing the informed consent form, they will be invited to participate in the trial, and the recruitment is expected to start in April 2025 and end when 200 participants are registered.

### Baseline assessment

The researchers will collect baseline information, including participants’ general personal data and demographic information such as gender, age, history of chronic medication use, etc., and will collect all observation indicators including Handgrip strength and 30-s sit-to-stand test.

### Randomization, assignment of hiding and blinding

The randomization sequence will be generated using computer-generated random numbers, and participants will be allocated to either the intervention or control group in a 1:1 ratio. Allocation concealment will be ensured by an independent third party utilizing opaque, sealed, sequentially numbered envelopes. The randomization operation was carried out by the staff of the GCP Center of Xiyuan Hospital of China Academy of Chinese Medical Sciences. Although blinding is difficult to implement in this trial, data analysts and result evaluators will follow the blinding principles when processing the data. In addition, objective measures were chosen as the primary outcome measures in this trial to minimize measurement bias.

### Intervention protocol

#### Control group

HD, during the 12-week intervention period, subjects will receive structured health education and dietary guidance covering the etiology, pathogenesis, at-risk populations, exercise rehabilitation, and nutritional support related to sarcopenia. The educational content will systematically address the therapeutic benefits of exercise rehabilitation, along with specific implementation parameters including duration, frequency, intensity, modality selection, and safety precautions.

#### Intervention group

BHD, guidance on the basis of health education and dietary guidance, during the intervention period, the research team will implement a 12-week standardized BDJ curriculum at fixed teaching sites (comprising one 70-min centralized session weekly). The course will adopt a four-module framework: biomechanical analysis of BDJ movements, consolidation and error correction of acquired techniques, new movement instruction, and group practical guidance. Dietary education and health guidance will be systematically integrated throughout the intervention process. After completing nine BDJ foundational training (including preparatory postures), participants will advance to movement trajectory optimization and respiratory rhythm regulation phases, designed to enhance movement precision. Subjects will perform standardized home-based training using instructional videos, completing daily 30-min BDJ practice sessions (4 times/week), combined with centralized sessions to achieve a weekly training frequency of 5 sessions (1 centralized + 4 home-based). A dual-time-point video documentation protocol (pre- and post-intervention) will be employed to archive visual records for instructional standardization control and movement quality evaluation.

During the trial, no necessary routine medical treatment was ruled out for either group of subjects. If drugs such as growth estrogen and testosterone were used to improve sarcopenia, they should be reported to the researchers, and the researchers should record the medication situation of the subjects in a timely manner.

### Outcome measure

In the trial, the outcome measures will be measured before the intervention begins, after the intervention ends, and at the follow-up. All measures will be conducted by professionally trained researchers, as shown in Figure 2 for specific time points and measures.

#### Primary outcome measures

##### Handgrip strength test and 30-s sit-to-stand test

Handgrip strength test (23) is a commonly used functional assessment method in clinical practice. It measures the maximum force produced by isometric contractions of the flexor muscle groups in the forearm of the subject with a standardized handheld dynamometer, and the test results objectively reflect the functional status of the upper limb skeletal muscles and neuromuscular coordination.

30-s sit-to-stand test (24) is a standardized test for assessing lower limb functional ability, requiring subjects to complete as many sit-to-stereoscopic position changes as possible within 30 s without upper limb assistance, reflecting quadriceps muscle strength, hip flexor coordination and dynamic balance ability through counting, and is widely used in geriatric functional assessment and sports injury rehabilitation monitoring.

#### Secondary outcome measures

Muscle electrophysiological test (25): an objective examination method that quantitatively assesses motor unit recruitment patterns, neuromuscular junction conduction efficiency, and muscle excitation-contraction coupling function by recording the electrical signal characteristics of muscles at rest and contraction through surface electrodes. This technique can specifically identify neurogenic and myogenic impairments to distinguish the pathological changes of sarcopenia in old age.

Soft tissue tension (26–28): refers to the intrinsic stress characteristics of connective tissues such as skeletal muscles, fascia, and ligaments at rest, reflecting the dynamic balance between neuromuscular regulatory mechanisms and the viscoelastic properties of the tissues. Abnormal increases and decreases are significantly associated with the pathological state of muscle.

6-min walk test (6MWT) (29): a standardized submaximal exercise test for assessing cardiopulmonary reserve. By measuring the maximum distance that the subject can walk independently within a specified time, it comprehensively reflects cardiopulmonary endurance, peripheral muscle function, and self-efficacy. It is the core objective indicator for the evaluation of the therapeutic effect of cardiopulmonary rehabilitation.

Musculoskeletal ultrasound (26, 30): imaging means for visual assessment. It can precisely identify tissues such as muscles, tendons, and synovial membranes of joints, enabling measurements of muscle dimensions.

Balance function assessment (31, 32): it is often used as part of the assessment of physical function in sarcopenia patients, it is aneuromotor control test system that quantitatively assesses vestibule-visual-proprioceptive integration ability through pressure center trajectory analysis combined with dynamic posture maps. This test is crucial for evaluating the risk of falls in older adults.

Quality of life assessment (QoL) (29, 33, 34): it is a 36-item universal health-related quality of life assessment tool that quantifies an individual’s multi-dimensional health status through eight dimensions, including physical function and body pain. It has high clinical validity in the assessment of chronic disease burden.

Short Physical Performance Battery (SPPB) (35): a standardized tool for assessing lower limb function in the older population, scores through three indicators: static balance maintenance, 4-meter walking speed, and five sit-down test times. Each 1-point reduction in the total score is significantly associated with an 8–12% increase in all-cause mortality, and it has a high predictive effect in aging frailty screening.

Body composition analysis (36): a metabolic assessment technique that quantitatively evaluates body fat percentage, skeletal muscle mass index and extracellular water ratio using bioelectrical impedance analysis (BIA). It has core application value in sarcopenia screening.

### Adverse events

The common adverse events of traditional exercise training are symptoms such as chest tightness, shortness of breath, limb pain, etc. The researcher must record in detail, including the time of occurrence of adverse reactions, symptoms, signs, severity and frequency of occurrence, duration of termination, treatment methods and results, course, follow-up time, recovery date, etc. Whether treatment is needed, if so, please record the treatment given. The investigator determines whether adverse events are related to the training program and provides evidence to support that judgment. When adverse events are identified, researchers can take necessary measures based on the condition. In the event of a serious adverse event, the unit undertaking the clinical study must immediately take necessary measures to protect the patient’s safety. Record in detail the process and outcome of the treatment until it is properly resolved or the patient’s condition is stable. If a Serious Adverse Event (SAE) occurs, the investigator must report it to the research group within 24 h or no later than the second working day, and report it to the ethics committee. The investigator should sign and date the report, and record in the original data when, in what manner, and to whom the serious adverse event was reported.

### Data recording, statistics and analysis

#### Data recording

The case report form (CRF) was filled out by the investigator, and each enrolled case had to complete the CRF. All cases were observed and treated according to the study protocol, and the medical record report forms were carefully filled out in accordance with the requirements for the study medical record. The subjects’ indicators were recorded truthfully. The CRF shall be kept as the original record and shall not be altered. The original record shall not be altered when any correction is made. Only additional statements shall be used to explain the reasons, signed by the physician participating in the clinical study and dated. The completed CRFs, after being reviewed by the monitor, are handed over to the data administrator for data management.

### Statistical analysis

Data will be analyzed by independent statisticians using the SPSS 27.0 software package. The primary analysis will adhere to the intention-to-treat (ITT) principle, supplemented by per-protocol (PPS) analysis, with a two-sided *p < 0.05* defined as statistical significance. Missing data will be addressed using multiple imputation methods. Continuous variables will be described as mean ± standard deviation (SD) or median (interquartile range, IQR), and categorical variables as frequencies (percentages). Baseline characteristics between the intervention group and the control group will be compared using *independent-samples t*-tests (for normally distributed data) or *Mann–Whitney U* tests (for non-normally distributed data). Categorical variables will be analyzed using chi-square tests or Fisher’s exact tests. Primary and secondary outcomes will be evaluated using mixed-effects linear models with restricted maximum likelihood (REML). The models will include group (Baduanjin/control), time (baseline/post-intervention), and other potential confounding factors as covariates.

### Dissemination

The study protocol is in line with the *Declaration of Helsinki* and is available through the International Traditional Medicine Clinical Trial Registry (itmctr.ccebtcm.org.cn). The results of the study will be published in a peer-reviewed scientific journal or at a local, national or international conference. The results of the study will also be shared directly with all participants and disseminated to researchers, health service providers, health care professionals and the general public through courses, presentations and the Internet.

## Discussion

BDJ, as a traditional body and mind exercise of light to moderate intensity, is designed to emphasize symmetry, coordination and breathing regulation, which can effectively activate the core muscles and promote the balanced development of muscles throughout the body (37–40). In addition, the low-intensity nature of BDJ makes it more suitable for older adults with reduced muscle mass and weakened muscle strength (41),reducing the risk of sports injuries while improving exercise compliance (42).

In terms of observation indicators, we not only selected the classic screening and diagnostic indicators for sarcopenia in older adults (43), but also selected more refined indicators including exercise endurance, balance function, subjective scales, etc. To comprehensively evaluate the rehabilitation effect of BDJ on older adult patients with sarcopenia. In order to provide clinical evidence for the rehabilitation of sarcopenia in older adults under the guidance of traditional exercises in the future.

This study plans to explore the intervention effect of BDJ exercise on older adult patients with sarcopenia through a randomized controlled trial (RCT). However, this study also has some limitations. Firstly, although we managed to control bias as much as possible through randomization and blinding design, due to the particularity of the exercise intervention, complete blinding could not be carried out on the participants and the intervention implements, which might have had some impact on the results. Secondly, other daily activities of the participants during the intervention were difficult to monitor completely, and the intensity of their activities could not be precisely quantified. Nevertheless, the findings of this study will still have significant clinical and public health implications. BDJ, as a traditional exercise that is easy to learn and promote, can provide a safe and effective non-pharmaceutical intervention for older adults in the community, especially those with sarcopenia (44). Future studies could further expand the sample size, extend the intervention period, and explore the combined effects of BDJ with other interventions, such as nutritional supplementation and resistance training, in order to provide a more comprehensive scientific basis for the prevention and treatment of sarcopenia in older adults.

In conclusion, this trial will provide preliminary evidence of the effectiveness of BDJ exercise in improving muscle mass and physical function in older adults patients with sarcopenia, supporting its wide application in the health management of older adults in the community.
